# Supplementary material for: A deep single cell mass cytometry approach to capture canonical and noncanonical cell cycle states
Source: Nat Commun. 2025 Oct 3;16:8821. doi: 10.1038/s41467-025-63883-4 (PMC12494979; doi:10.1038/s41467-025-63883-4)
Supplement: Supplementary file 1 — Supplementary Information [file 41467_2025_63883_MOESM1_ESM.pdf]

Supplementary Information
Amouzgar et al. 2025

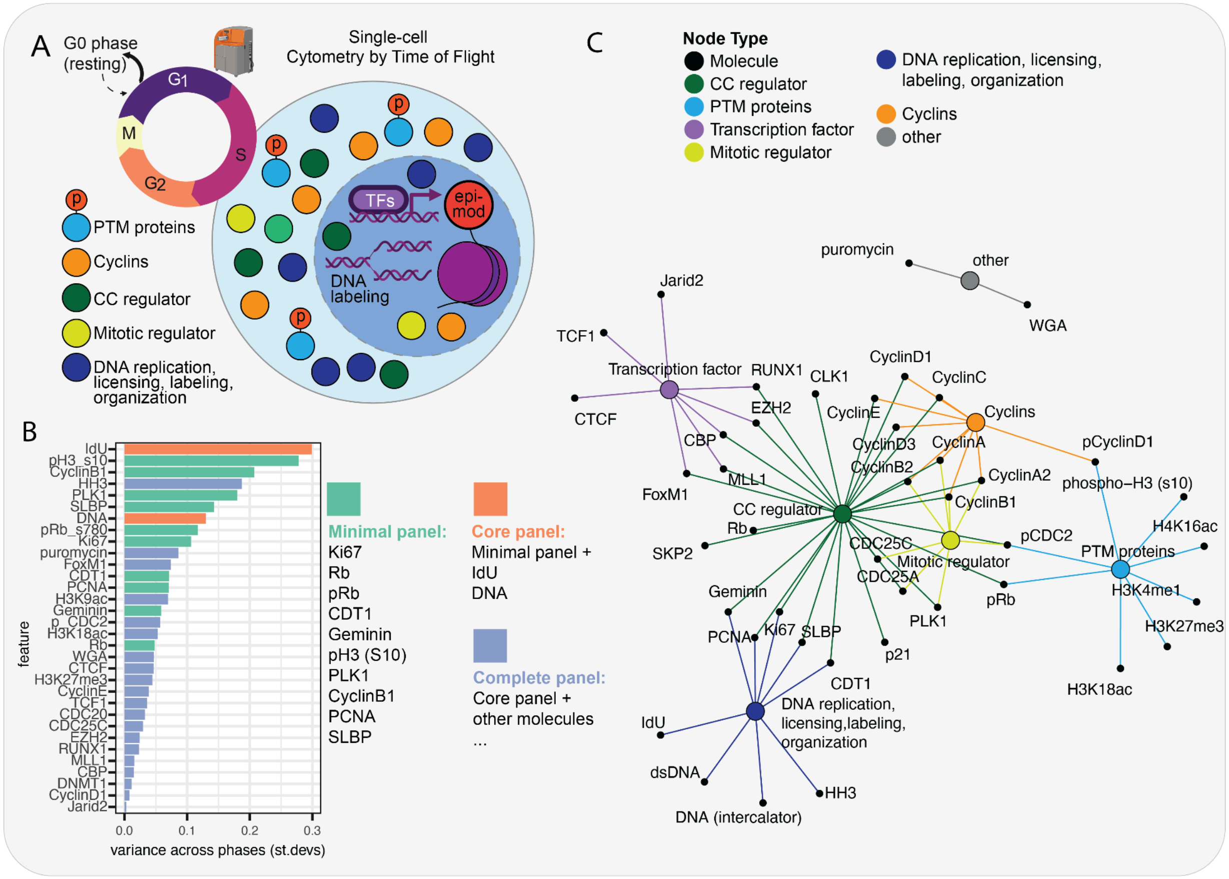

Supplementary Figure 1: Cartoon diagram of CC related molecules mapped to different categories

(A) Measured CC molecules are mapped to different categories. CC regulator=direct control of CC progression. PTM proteins=post translational modification. Mitotic regulator=direct control of G2M transition or mitotic progression. DNA replication, licensing, labeling, organization=measurements relating to DNA replication during S phase, DNA replication licensing, nucleotide labeling, intercalation, or histone content. Cyclins=Cyclin molecule, phosphorylated or not. Transcription factor=Any molecule also categorized as a transcription factor. Other=WGA for cell size, and puromycin for de novo protein synthesis measured using puromycin incorporation. **(B)** Feature variance across cell cycle phases to detect most variable targets for separating cell cycle progression. **(C)** Graphical diagram of features and category mapping.

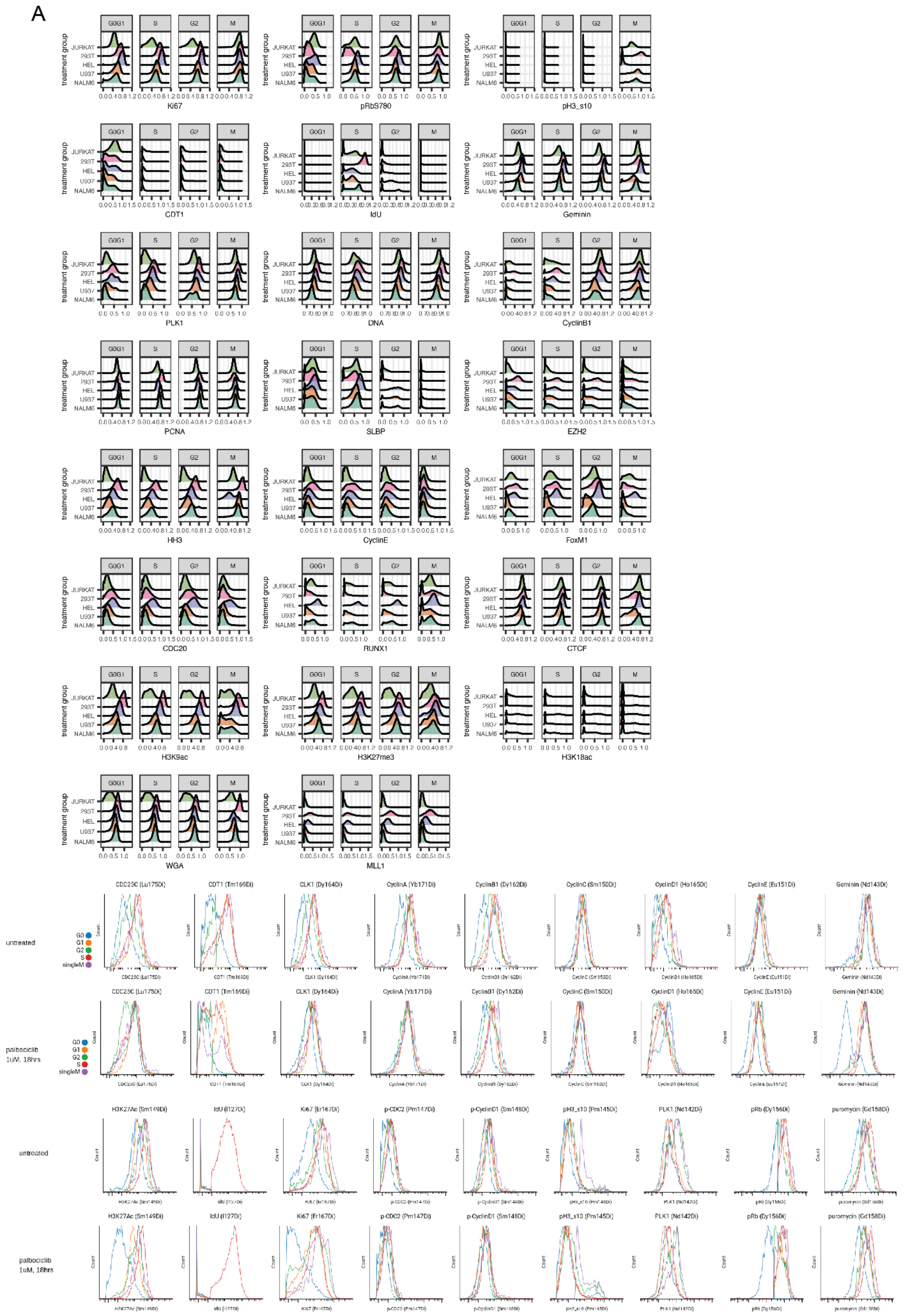

Supplementary Figure 2: Molecular abundance of CC related targets across cell lines
(A) *Smooth histograms of example CC targets across cell lines and drug treatments (1 representative sample).*

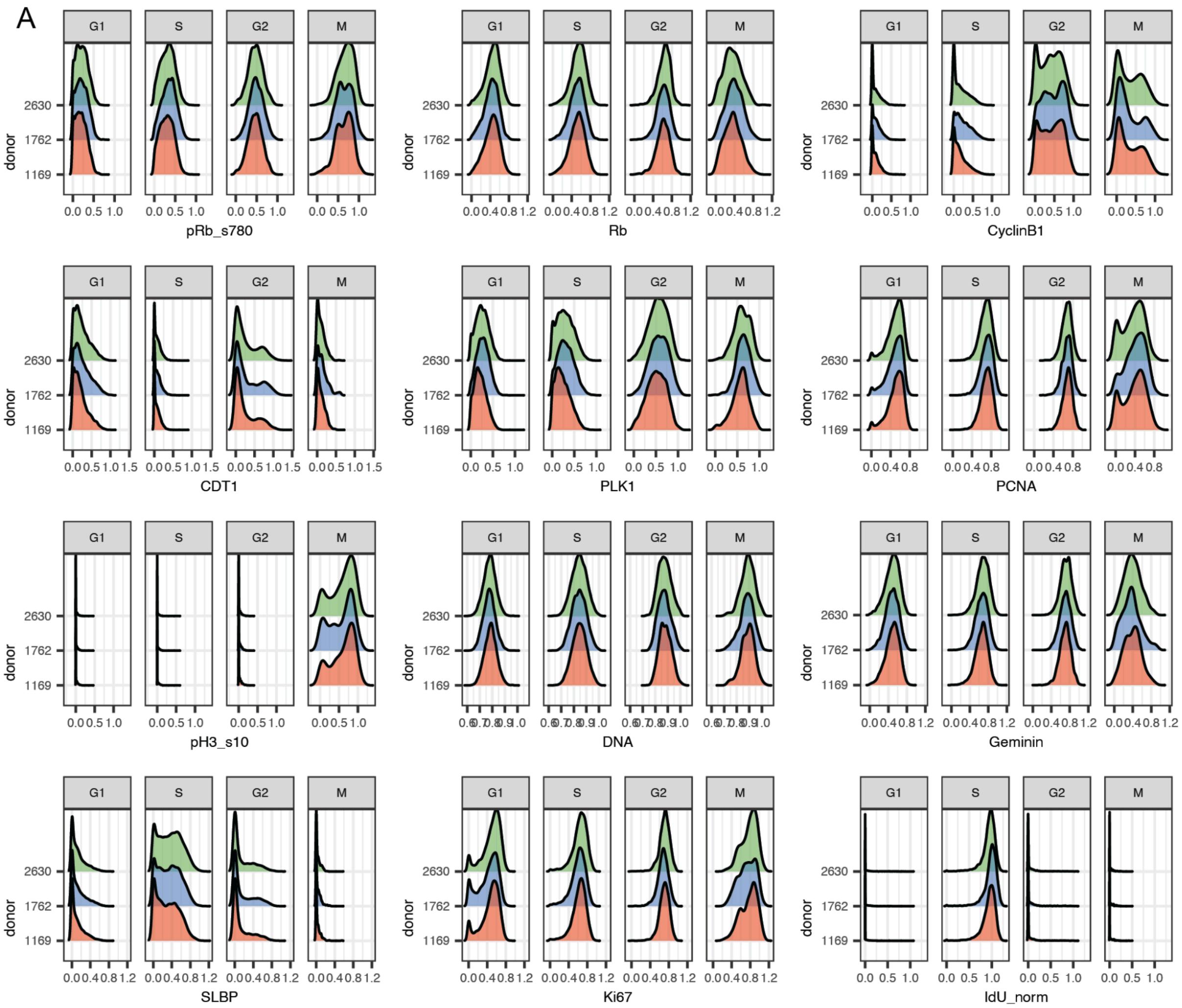

Supplementary Figure 3: Molecular abundance of core CC targets across 3 primary human T cells from 3 donors. ***(A)*** *Smooth histograms of CC markers across each donor (n=3).*

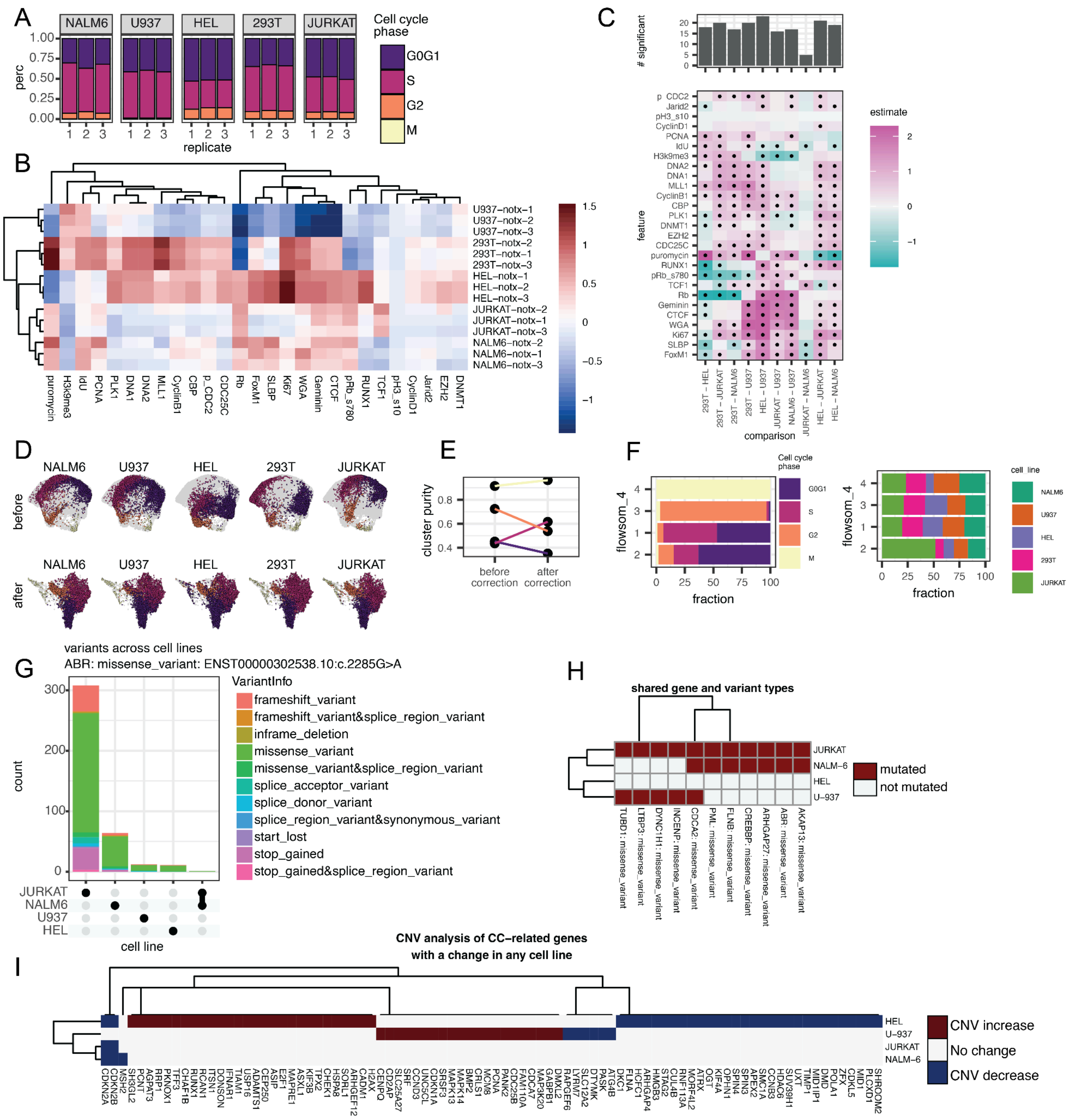

Supplementary Figure 4: Canonical and noncanonical CC states across cell lines
(A) *CC fractions across different cell line replicates (n=3 each).* ***(B)*** *Hierarchically clustered heatmap of mean cell cycle molecular abundance across cell line replicates.* ***(C)*** *Differential abundance analysis of all pairwise cell lines for different cell cycle related markers with # of significant features (barplot), scaled model coefficient estimate aka change in abundance (heatmap), and significance labels defined by padj<=0.1 and estimates >= 0.25 (points) using generalized linear models and the Wald test.* ***(D)*** *CC embedding before and after integrating cell lines using harmony.* ***(D)*** *CC phase purity analysis of clustering before and after correction.* ***(E)*** *FlowSOM with 4 clusters colored by cell cycle phase (top) and* ***(F)*** *Fraction of each cell line in each cluster (equally sampled across cell lines, bottom).* ***(G-I)*** *Variant analysis comparing CC genes for cell lines across three scales of variant information available for Jurkat, HEL, U-937, and NALM-6 but not HEK293T in the DepMap database: (1) exact SNV (most specific), (2)*

variant type in a gene regardless of specific nucleotide position (eg, a missense variant in ABR regardless of specific SNV), and (3) CNVs. ***(G)*** *Upset plot of exact SNVs across different cell lines. 1 unique missense SNV shared between Jurkat and NALM-6 cells: a Guanine to Adenine nucleotide change in ABR at ENST00000302538.10:c.2285G>A.* ***(H)*** *Binary heatmap of variant type in a gene regardless of specific nucleotide position.* ***(I)*** *Heatmap of genes binned for increased, no change, or decreased CNVs detected in a cell line.*

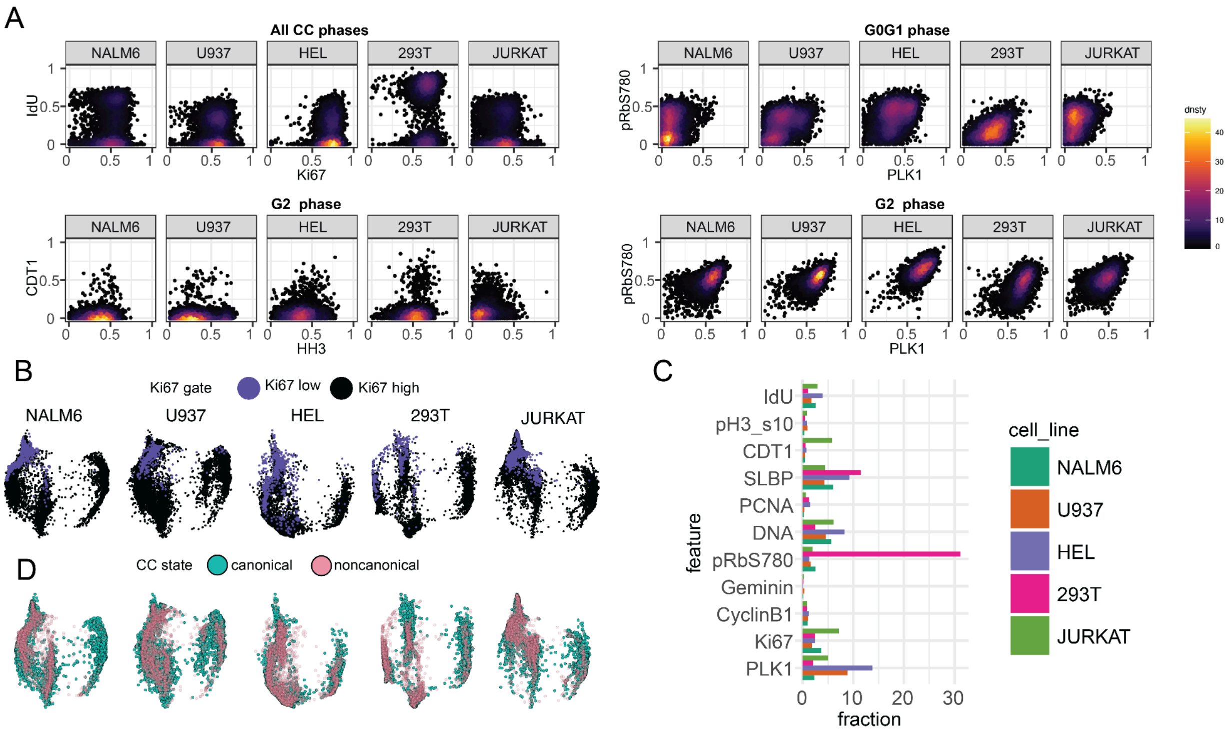

Supplementary Figure 5: Canonical and noncanonical CC states across cell lines
(A) *Flow plots with example markers for phases with noncanonical states.* ***(B)*** *CC embeddings for each cell line colored by Ki67 low abundance.* ***(C)*** *fraction of cells with noncanonical identities based on manual discretization in each cell line.* ***(D)*** *CC embeddings for each cell line colored by canonical or noncanonical state.*

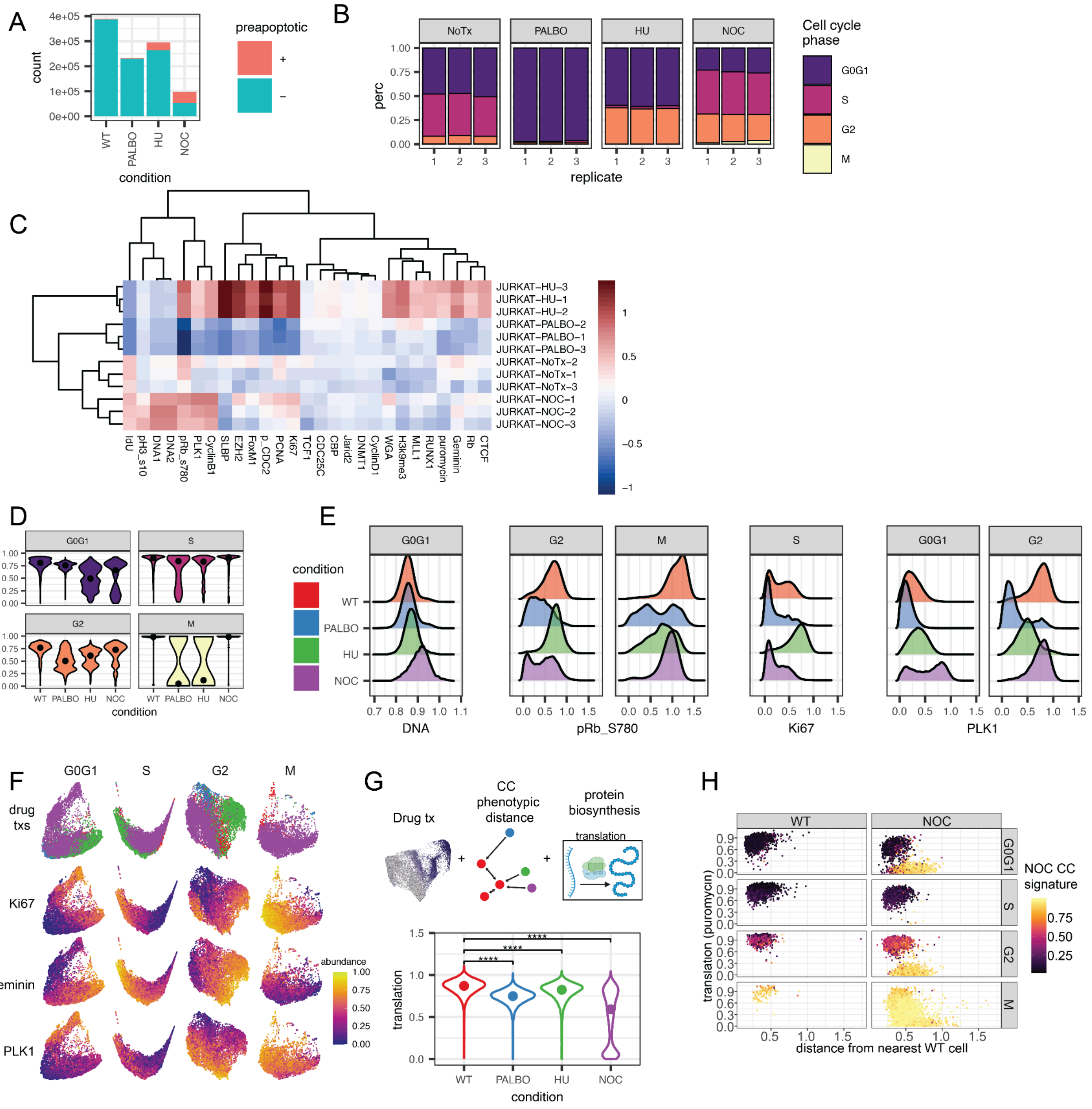

Supplementary Figure 6: Drug perturbation effects

***(A)** Removal of cPARP+ pre-apoptotic cells. # of cells before gating; WT: 390074, PALBO: 232619 , NOC: 97727, HU: 295362. Remaining normal cells after removal: WT: 386,643, PALBO: 229,629, HU: 263,428, NOC: 53,129. **(B)** Cell cycle fractions for 3 replicates of each drug in Jurkat cells. CC embeddings faceted by treatment condition. **(C)** Heatmap of molecular abundance for all replicates. **(D)** Cell cycle phase scores. **(E)** Smoothed histograms for example markers of noncanonical cells. **(F)** CC embeddings of canonical and noncanonical cells computed on each group with example markers. **(G)** Cartoon schematic integrating drug perturbations with phenotypic distance analysis for noncanonical CC states and protein biosynthesis measurements using MC and drug perturbation. Two-sided Wilcoxon rank sum*

*test, p-values: * <=0.05, **<=0.01, ***<=0.001).* ***(H)*** *Relationship between protein translation, phenotypic distance, and NOC signature for NOC-treated and WT cells.*

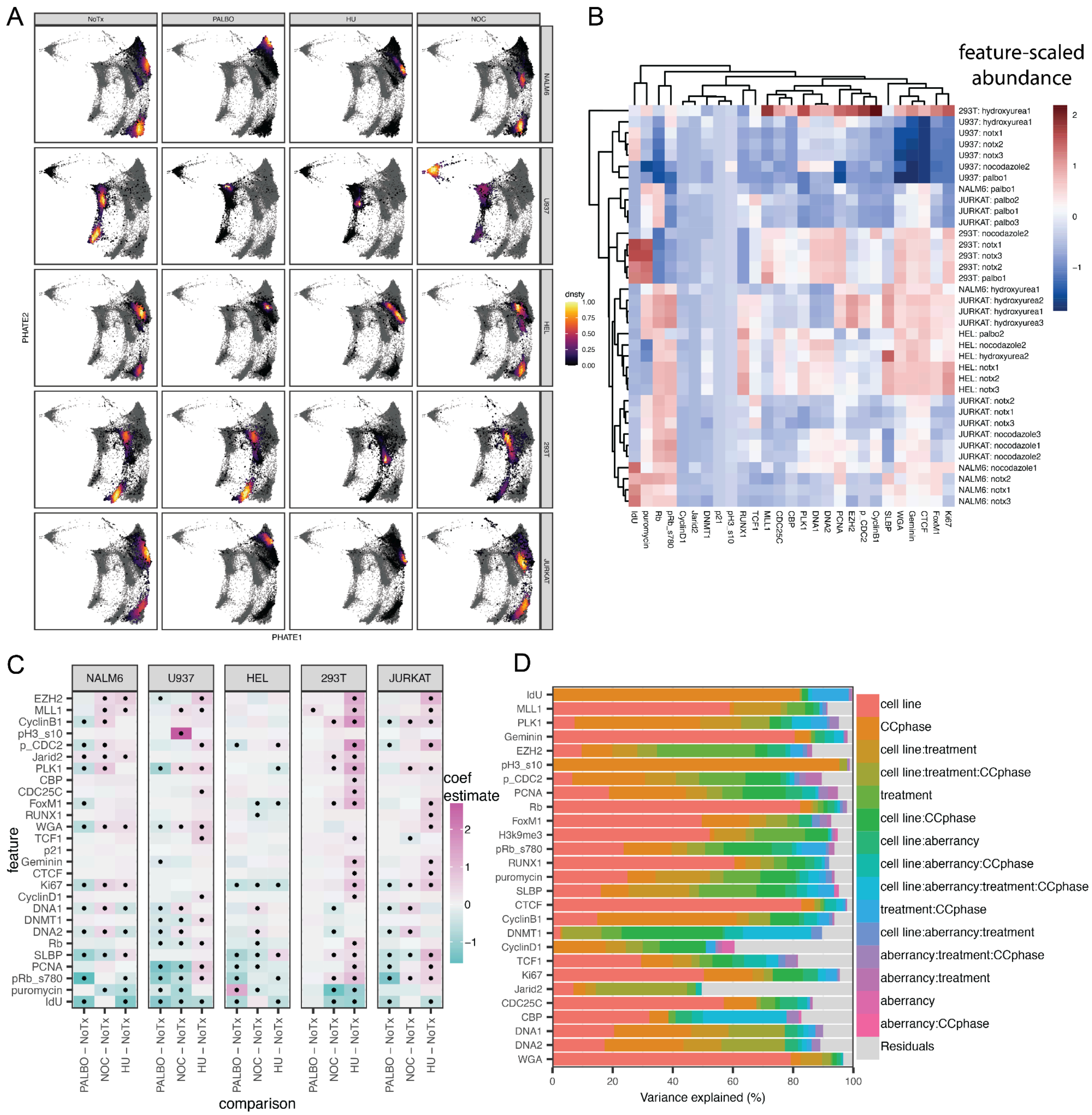

Supplementary Figure 7: Drug perturbation effects

(A) *CC embedding of cell lines from NoTx, Palbociclib, Hydroxyurea, and Nocodazole treatments.* ***(B)*** *Heatmap of all replicates for cell lines from NoTx, Palbociclib, Hydroxyurea, and Nocodazole treatments.* ***(C)*** *Heatmap of coefficient estimates (fold-change) comparing drug treatment to no treatment in each cell line. Significant features are detected with an adjusted-pvalue threshold <=0.1, and minimum absolute value coefficient (fold-change) threshold of 0.25 using generalized linear models and the Wald test.* ***(D)*** *Percentage of variance explained for all main and interaction effects in each feature.*

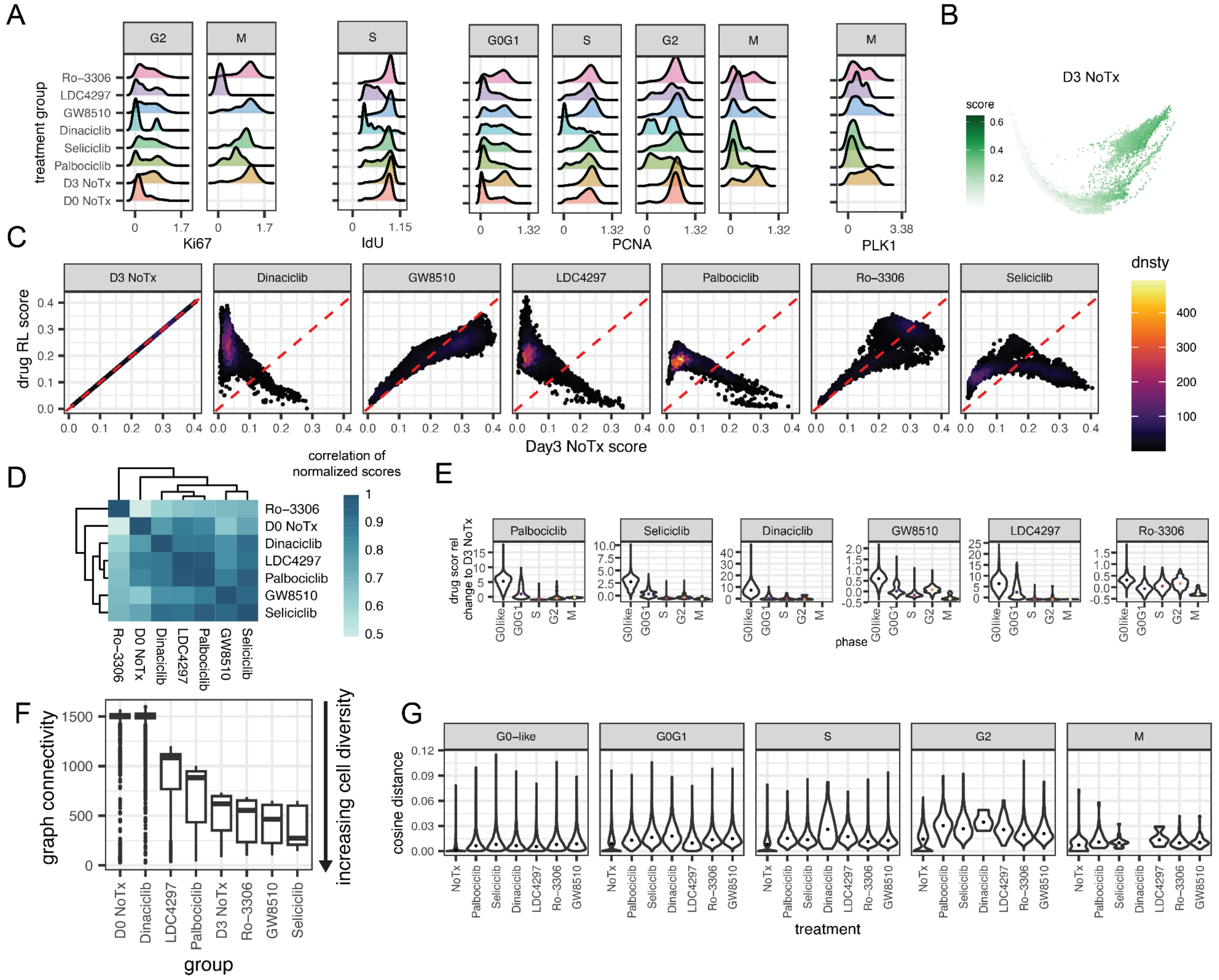

Supplementary Figure 8. CC inhibitor action on ex vivo stimulated primary human T cells
(A) *Expression of example CC targets for each condition in different groups.* ***(B)*** *CC embedding colored by D3 NoTx score.* ***(C)*** *D3 NoTx likelihood score versus each drug's likelihood score.* ***(D)*** *Correlation between drug treatment effects.* ***(E)*** *Normalized drug likelihood score across each phase.* ***(F)*** *Cell diversity analysis for each treatment using graph connectivity analysis.* ***(G)*** *CC phenotypic distance for each treatment across each phase.*
