# Supplementary material for: A deep single cell mass cytometry approach to capture canonical and noncanonical cell cycle states
Source: Nat Commun. 2025 Oct 3;16:8821. doi: 10.1038/s41467-025-63883-4 (PMC12494979; doi:10.1038/s41467-025-63883-4)
Supplement: Supplementary file 2 — Description of additional supplementary files [file 41467_2025_63883_MOESM2_ESM.pdf]

# Description of Additional Supplementary Files

Supplementary Data 1. Tested molecular targets

Supplementary Data 2. SNV analysis from published DebMap data

Supplementary Data 3. CNV analysis

Supplementary Data 4. Example design matrix

Supplementary Data 5. Drug concentrations

Supplementary Data 6. Differential analysis results between cell lines from atlas data

Supplementary Data 7. Differential analysis results between treatments from atlas data

Supplementary Data 8. Variance Partition results from atlas data

Supplementary Data 9. Packages

Supplementary Data 10. Features used for dimensionality reduction
