# Supplementary material for: A deep single cell mass cytometry approach to capture canonical and noncanonical cell cycle states
Source: Nat Commun. 2025 Oct 3;16:8821. doi: 10.1038/s41467-025-63883-4 (PMC12494979; doi:10.1038/s41467-025-63883-4)
Supplement: Supplementary file 3 — Reporting Summary [file 41467_2025_63883_MOESM3_ESM.pdf]

nature portfolio

Corresponding author(s): Sean Bendall

Last updated by author(s): 2025-Aug-18

Reporting Summary

Nature Portfolio wishes to improve the reproducibility of the work that we publish. This form provides structure for consistency and transparency in reporting. For further information on Nature Portfolio policies, see our Editorial Policies and the Editorial Policy Checklist.

Statistics

For all statistical analyses, confirm that the following items are present in the figure legend, table legend, main text, or Methods section.

n/a	Confirmed	
☐	☒	The exact sample size (*n*) for each experimental group/condition, given as a discrete number and unit of measurement
☐	☒	A statement on whether measurements were taken from distinct samples or whether the same sample was measured repeatedly
☐	☒	The statistical test(s) used AND whether they are one- or two-sided<br>*Only common tests should be described solely by name; describe more complex techniques in the Methods section.*
☐	☒	A description of all covariates tested
☐	☒	A description of any assumptions or corrections, such as tests of normality and adjustment for multiple comparisons
☐	☒	A full description of the statistical parameters including central tendency (e.g. means) or other basic estimates (e.g. regression coefficient) AND variation (e.g. standard deviation) or associated estimates of uncertainty (e.g. confidence intervals)
☐	☒	For null hypothesis testing, the test statistic (e.g. *F*, *t*, *r*) with confidence intervals, effect sizes, degrees of freedom and *P* value noted<br>*Give P values as exact values whenever suitable.*
☒	☐	For Bayesian analysis, information on the choice of priors and Markov chain Monte Carlo settings
☒	☐	For hierarchical and complex designs, identification of the appropriate level for tests and full reporting of outcomes
☒	☐	Estimates of effect sizes (e.g. Cohen's *d*, Pearson's *r*), indicating how they were calculated

Our web collection on statistics for biologists contains articles on many of the points above.

Software and code

Policy information about availability of computer code

Data collection	Raw single-cell data was collected using the Fluidigm CyTOF software on a Helios system available through standardbio. No public data or custom software for data collection was used.
Data analysis	All data processing and analysis was performed using R v4.4.2 or Python 3.12.8 .<br>tidyverse 2.0.0<br>pROC 1.18.5<br>nnet 7.3.19<br>caret 7.0.1<br>patchwork 1.3.0<br>rstudioapi 0.17.1<br>magrittr 2.0.3<br>phateR 1.0.7<br>uwot 0.2.2<br>igraph 2.1.1<br>stats 4.4.2<br>MASS 7.3.61<br>meld 1.0.2<br><br>Further relevant details are provided in the methods section. No custom code was used to analyze the data in this study. Machine learning code and example data used in this study is available on the github link and provided in the ML checklist form. https://github.com/

mamouzgar/2025_cellcycle_ml

For manuscripts utilizing custom algorithms or software that are central to the research but not yet described in published literature, software must be made available to editors and reviewers. We strongly encourage code deposition in a community repository (e.g. GitHub). See the Nature Portfolio guidelines for submitting code & software for further information.

Data

Policy information about availability of data

All manuscripts must include a data availability statement. This statement should provide the following information, where applicable:

- Accession codes, unique identifiers, or web links for publicly available datasets
- A description of any restrictions on data availability
- For clinical datasets or third party data, please ensure that the statement adheres to our policy

All data (fcs files) are uploaded to a Zenodo repository (10.5281/zenodo.14852934) that is ready for public release without any restrictions. This link will be accessible via: https://doi.org/10.5281/zenodo.14852934

Research involving human participants, their data, or biological material

Policy information about studies with human participants or human data. See also policy information about sex, gender (identity/presentation), and sexual orientation and race, ethnicity and racism.

Reporting on sex and gender	Not applicable because deidentified donor samples were purchased from the Stanford Blood Center and this information is inaccessible.
Reporting on race, ethnicity, or other socially relevant groupings	Not applicable because deidentified donor samples were purchased from the Stanford Blood Center and this information is inaccessible.
Population characteristics	See above
Recruitment	Not applicable because deidentified donor samples were purchased from the Stanford Blood Center and this information is inaccessible.
Ethics oversight	Not applicable because deidentified donor samples were purchased from the Stanford Blood Center and this information is inaccessible.

Note that full information on the approval of the study protocol must also be provided in the manuscript.

Field-specific reporting

Please select the one below that is the best fit for your research. If you are not sure, read the appropriate sections before making your selection.

- [x] Life sciences
- [ ] Behavioural & social sciences
- [ ] Ecological, evolutionary & environmental sciences

For a reference copy of the document with all sections, see nature.com/documents/nr-reporting-summary-flat.pdf

Life sciences study design

All studies must disclose on these points even when the disclosure is negative.

Sample size	No calculation was performed to determine sample sizes. Experiments were analyzed with 3+ replicates or donors where applicable. Cell numbers were collected in excess (100,000 or more) cells to ensure cell heterogeneity is captured.
Data exclusions	Pre-apoptotic cells (cPARP positive) were removed from the analysis. Other general preprocessing strategies to exclude cells or debris across any experiment (eg, event length, debris and doublet removal, etc) are described in figure 1 and the methods.
Replication	Analysis was performed in replicates of three from different donors/samples.
Randomization	Randomization was not relevant to this study as there were no clinical or in vivo studies.
Blinding	Blinding was not relevant to this study as there were no clinical or in vivo studies.

Reporting for specific materials, systems and methods

We require information from authors about some types of materials, experimental systems and methods used in many studies. Here, indicate whether each material, system or method listed is relevant to your study. If you are not sure if a list item applies to your research, read the appropriate section before selecting a response.

Materials & experimental systems

n/a	Involved in the study
	☒ Antibodies
	☒ Eukaryotic cell lines
☒	☐ Palaeontology and archaeology
☒	☐ Animals and other organisms
☒	☐ Clinical data
☒	☐ Dual use research of concern
☒	☐ Plants

Methods

n/a	Involved in the study
☒	☐ ChIP-seq
☒	☐ Flow cytometry
☒	☐ MRI-based neuroimaging

Antibodies

Antibodies used

A detailed table of all antibodies tested including clone numbers, vendor, and concentrations are provided in the supplementary table.

PLK1 abcam
Geminin abcam
phospho-H3 (s10) biolegend
H3K18ac CST
Rb bd biosciences
phospho-Rb bd biosciences
SLBP abcam
CDT1 CST
CDT1 CST
CyclinB1 bd biosciences
Ki67 biolegend
PCNA Thermo Fisher Scientific
PCNA Thermo Fisher Scientific
cPARP bd biosciences
CLK1 sc-biotech
HH3 CST
EZH2 CST
p-CDC2 CST
pCyclinD1 CST
puromycin EMD Millipore
FoxM1 CST
pRb bd biosciences
CyclinA biolegend
cCasp3 CST
CyclinD1 abcam
SKP2 abcam
CyclinB2 abcam
CDC25A sc-biotech
CDC25C sc-biotech
CyclinD1 novus biologicals
CyclinC CST
CyclinE sc-biotech
CyclinD3 abcam
CyclinB1 bd biosciences
DNA (intercalator) -
IdU -
WGA -
CyclinA2 BioLegend
CyclinB BD
CyclinD1 CST
CyclinA abcam
CyclinB1 BD
CyclinD1 abcam
p-21-Waf1/Cip1 CST
phospho-Rb CST
CTCF CST
RUNX1 bioLegend
H3K27me3 CST
Cyclin A sc-biotech

	H4K16ac CST<br>H3K4me1 CST
Validation	In addition to selecting for antibodies with supplier-based antiody validation based on FCM validation provided on the vendor's website, further in-house experimental validation was performed using expected gates on expected negative and positive populations observed in non-proliferating/proliferative cells, specific cell cycle phases, or as a consequence of cell cycle perturbation to observe a decrease in molecule expression in non-cycling (cells (eg, unactivated T cells or arrested cells) compared to cycling cells, or through deeper in silico gating of cell cycle phases and comparing expression across phases, such as comparing molecule expression in G0G1 to G2 phase.

Eukaryotic cell lines

Policy information about cell lines and Sex and Gender in Research

Cell line source(s)	Cell lines are sourced from ATCC
Authentication	No authentication for cell lines was performed.
Mycoplasma contamination	No cell lines were tested for mycoplasma contamination.
Commonly misidentified lines (See ICLAC register)	n/a

Plants

Seed stocks	n/a
Novel plant genotypes	n/a
Authentication	n/a
